# Cohort study protocol: Bioresource in Adult Infectious Diseases (BioAID)

**DOI:** 10.12688/wellcomeopenres.14690.1

**Published:** 2018-08-08

**Authors:** Laura J. Shallcross, Alexander Mentzer, Saadia Rahman, Graham S. Cooke, Shiranee Sriskandan, Mahdad Noursadeghi

**Affiliations:** 1 Institute of Health Informatics, University College London, London, NW1 2DA, UK; 2 Wellcome Trust Centre for Human Genetics and the Jenner Institute, University of Oxford, Oxford, UK; 3 Biomedical Research Centre, Great Ormond Street Hospital for Children, London, WC1N 1EH, UK; 4 Department of Medicine, Imperial College London, London, SW7 2AZ, UK; 5 NIHR Health Protection Unit in Antimicrobial Resistance and Healthcare Acquired Infection, Imperial College London, London, UK; 6 Division of Infection and Immunity, UCL, London, UK; 7 National Insitute of Health Research Biomedical Research Centre, UCLH/UCL, London, UK

**Keywords:** bioresource; infectious diseases, emergency department, genomics, diagnostics, epidemiology, microbiology, bacterial infection

## Abstract

**Introduction:** Infectious diseases have a major impact on morbidity and mortality in hospital. Microbial diagnosis remains elusive for most cases of suspected infection which impacts on the use of antibiotics. Rapid advances in genomic technologies combined with high-quality phenotypic data have great potential to improve the diagnosis, management and clinical outcomes of infectious diseases.  The aim of the Bioresource in Adult Infectious Diseases (BioAID) is to provide a platform for biomarker discovery, trials and clinical service developments in the field of infectious diseases, by establishing a registry linking clinical phenotype to microbial and biological samples in adult patients who attend hospital with suspected infection.

**Methods and analysis:** BioAID is a cohort study which employs deferred consent to obtain an additional 2.5mL RNA blood sample from patients who attend the Emergency Department (ED) with suspected infection when they undergo peripheral blood culture sampling.  Clinical data and additional biological samples including DNA, serum and microbial isolates are obtained from BioAID participants during hospital admission.  Participants are also asked to consent to be recalled for future studies. BioAID aims to recruit 10,000 patients from 5-8 sites across England.  Since February 2014 >4000 individuals have been recruited to the study.  The final cohort will be characterised using descriptive statistics including information on the number of cases that can be linked to biological and microbial samples to support future research studies. Ethical approval and section 251 exemption have been obtained for BioAID researchers to seek deferred consent from patients from whom a RNA specimen has been collected. Samples and meta-data obtained through BioAID will be made available to researchers worldwide following submission of an application form and research protocol.

**Conclusions:** BioAID will support a range of study designs spanning discovery science, biomarker validation, disease pathogenesis and epidemiological analyses of clinical infection syndromes.

## Introduction

Adult infectious diseases have a major impact on morbidity and mortality in hospitals worldwide ^1– 3^. These trends are driven by demographic changes associated with an ageing population, widespread use of immunosuppressive therapies and complex surgery in routine healthcare, and the emergence of new and drug-resistant pathogens. Although the use of molecular diagnostics has brought advances in the management of infectious diseases ^4, 5^, for most cases of infection in hospital the microbial cause of unselected febrile illnesses remains elusive. Consequently empiric treatment decisions are based on clinical and epidemiological knowledge of infectious disease syndromes. Technological advances in genomics, transcriptomics, proteomics and metabolomics combined with high-quality data on clinical phenotype have great potential to improve the diagnosis and management of infectious diseases. Bio-resources have already been established for specific infections such as HIV and HCV ^6, 7^. There are no other studies, to our knowledge, that set out to recruit unselected patients presenting acutely to hospital with suspected infection. Few studies have investigated diagnosis of infection in the emergency department (ED) population ^8– 10^, reflecting the difficulty in obtaining samples and consent in the acute setting and this creates an imbalance in the ability to undertake research in the field of acute infection. Innovation in this field is contingent on access to high-quality clinical data linked to prospective collection of biological samples.

BioAID has been established as a registry of unselected patients who present to the ED with suspected infectious diseases. The Bioresource is part of the Department of Health’s National Institute for Health Research Bioresource Programme which provides a registry of healthy volunteers and patients who have been consented for recall by virtue of genotype or phenotype to participate in secondary studies.

## Methods and analysis

### Aim of the study

BioAID has established a network of UK hospitals which provide the infrastructure for infectious disease research by linking clinical phenotype to microbial and biological samples. This will support the development and evaluation of novel diagnostic and risk stratification tools based on the application of emerging technologies for biomarker discovery and the conduct of clinical trials and service developments. The Bioresource also provides a platform for studies which investigate the genetic and immunological basis for host susceptibility to infectious diseases, which is likely to have a bearing on vaccine development.

### Choice of study design

A cohort design with deferred consent was selected due to the need to sample patients prospectively, the difficulties associated with obtaining genuine informed consent in the ED, and our desire to sample an unselected group of patients with suspected infectious diseases. Clinical data were extracted by a combination of medical note review and extraction of data from electronic health records deemed to be the most efficient and cost-effective method to obtain detailed and reliable information from a large number of participants across multiple sites.

### Study sites

BioAID was originally established in 2014 in London across five National Institute of Health Research (NIHR) Comprehensive Biomedical Research Centres (BRC’s). To date, patients are being recruited at two centres (University College London Hospital (UCLH) and Imperial College Healthcare NHS Trust, ICHT, the latter comprising three main sites with two EDs. Queen Elizabeth Hospital Birmingham (QEHB) will join BioAID in 2018 establishing a site outside of London. The proportion of patients who have blood culture sampling in the ED and are subsequently recruited to BioAID is estimated to be 15–20% across the ICHT sites and UCLH and the majority of these patients have been admitted to hospital. Both sites are London teaching hospitals which serve urban and ethnically diverse populations. The first samples were collected from individuals at UCLH in February 2014.

### Patient recruitment and consent

**Figure 1.**
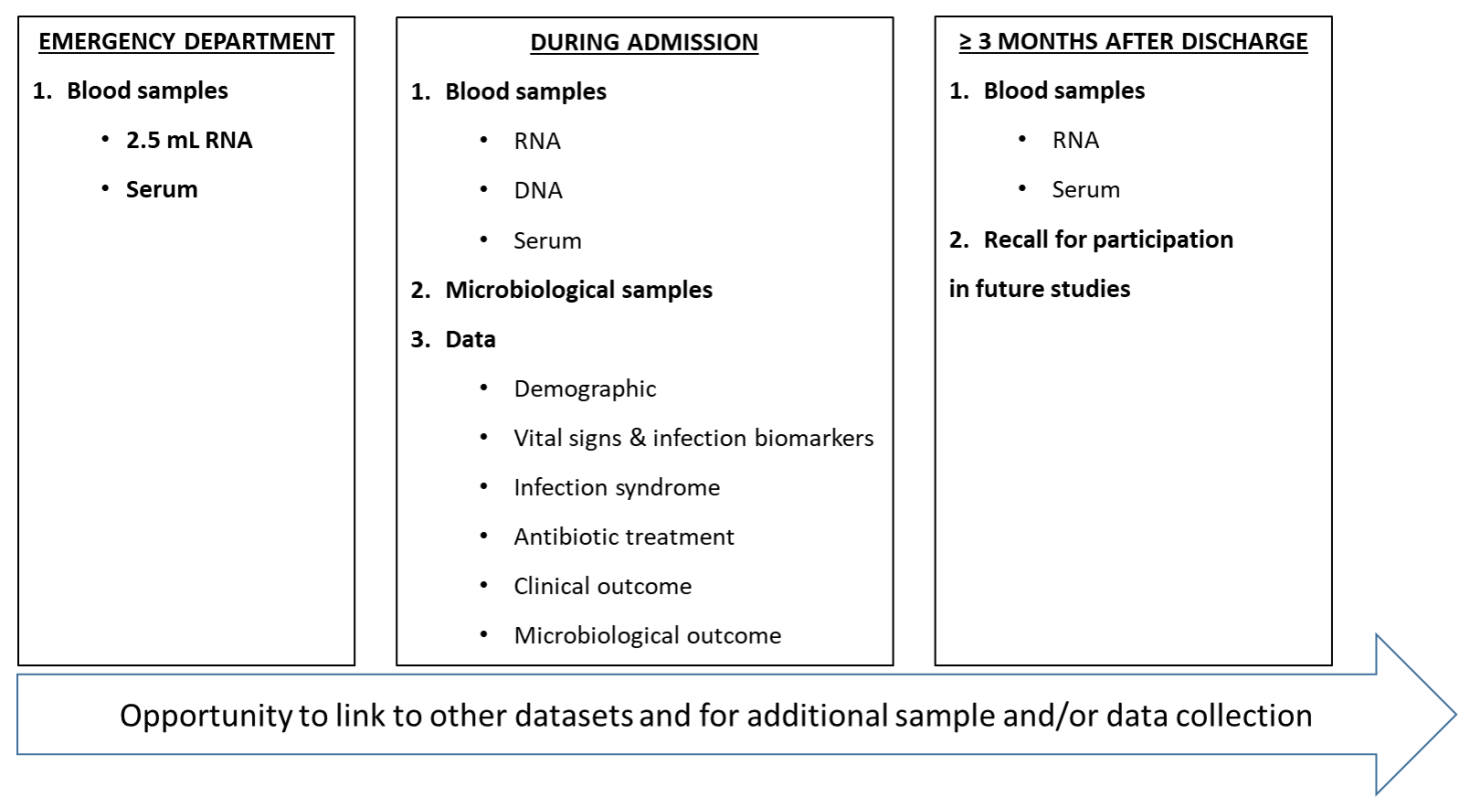
Collection of data and samples in BioAID.

Individuals aged > 16 years with suspected infection are eligible for inclusion in BioAID provided they undergo peripheral blood sampling for microbial culture, which is part of routine clinical assessment, contemporaneous with collection of an additional 2.5mL RNA blood sample in the ED, Figure 1. Within the following 72 hours, clinical research staff approach all participants from whom a RNA sample has been collected in order to invite them to take part in the study, provide detailed information on the study, and obtain informed written consent. Informed consent is sought by telephone from patients who have not been admitted to hospital, but have provided a RNA sample, as well as those who have been admitted but discharged before consent could be obtained. Importantly, informed consent is also sought by telephone from the next of kin in the case of patients who have died but have provided a RNA sample at the time of blood cultures being drawn. Consent is also sought for participants to be recalled to participate in future research studies. The acceptability of this approach was investigated at University College London Hospital in the FEVER study ^11^, and was deemed to be acceptable to both patients and their relatives.

### Collection and retrieval of biological samples

Serum samples obtained as part of routine care at the time of admission, but surplus to diagnostic requirements are also retrieved for the BioAID collection. DNA and additional RNA sample are collected during the patient’s admission to hospital, usually within 72 hours ( Table 1). RNA samples will be used primarily to evaluate host responses to infection at the transcriptional level. The paired RNA samples will be used to investigate whether the timing of the sample impacts on blood transcriptional profiles. Serum samples will be available to evaluate host responses to infection by proteomic and metabolomic profiling, as well as serological assays and quantitation of cytokine responses. DNA extraction provides the opportunity for future research studies investigating host genetic variants associated with the response to infection. Microbial isolates derived from specimens obtained during admission are retrieved from the laboratory and stored, laying the foundation for future studies of infection surveillance, diagnosis, pathogen evolution and genomics, disease pathogenesis and host-pathogen interaction. Participants are invited to attend the hospital for a follow-up visit three months after admission when convalescent serum and RNA samples may also be obtained.

**Table 1.** Overview of the sampling in BioAID.

Time since admission	Blood sample	Volume	Use
Presentation	RNA preservation tube	2.5mL	RNA extraction
	Serum (clot activator)	2-4mL	Serum analytes Acute serum
Throughout hospital stay	Various microbiological samples	Urine, Stool Throat swab, sputum etc.	Microbe identification
Within 1 week of presentation	RNA preservation tube	2.5mL	RNA extraction
	PAXgene / EDTA	8.5mL/10mL	DNA extraction
3 months after presentation	RNA preservation tube	2.5mL	RNA extraction
	Serum (clot activator)	5mL	Convalescent serum

### Handling of biological samples

RNA samples are collected in Tempus ^TM^ tubes and transferred to the microbiology laboratory with the blood culture samples. If deferred consent is obtained, the RNA sample is re-labelled with a study identifier in place of the patient identification label. If informed consent is not provided specimens are destroyed. All RNA and DNA samples are labelled with the study identifier and stored at -80°C. Microbial isolates and sera obtained from the patient during hospital admission are retrieved from the laboratory, re-labelled with the study identifier and stored at -80°C. All participants and samples are registered in the Bioresource database.

### Extraction of clinical data from the medical records

A standardised case reporting form ( Supplementary File 1) is used by a member of the clinical research team at each site to extract data from the medical record and/or data are extracted directly from the electronic medical record. This includes demographic information (age, gender, ethnicity); vital signs and clinical scores at admission; laboratory biomarkers for infection; syndrome of infection; antibiotic treatment; microbiological culture results and sensitivities for blood and other samples; and clinical outcome (duration of hospital admission, ICD-10 coded diagnosis on discharge, admission to intensive care, death). Sample type is classified according to the microbiology bench where the sample was processed. Antibiotic treatment was classified according to the antimicrobial section of the British National Formulary and microbiological culture results are classified by species.

Data are transcribed by the research team into the research database which has been developed using REDcap electronic data capture tools hosted at UCLH and ICHT. REDCap (Research Electronic Data Capture) is a secure, web-based application designed to support data capture for research studies providing 1) an intuitive interface for data entry; 2) audit trails for tracking data manipulation and export procedures; 3) automated export procedures for seamless data downloads to common statistical packages; and 4) procedures for importing data form external sources ^12^.

### Statistical analysis and sample size

BioAID aims to recruit 10,000 patients over 8 years across 5–8 participating centres. These estimates are based on pilot data from the FEVER study at UCLH ^11^. Novel genetic variants associated with infection related phenotypes have commonly required sample sizes ranging from 1000–5000 individuals in order to detect clinically meaningful associations between variant and phenotype ^13^. Larger sample sizes would be expected to enable detection of smaller genetic effects (risk ratios < 2.0). This Bioresource would therefore be expected to contribute to such studies assessing susceptibility to infection or survival following sepsis. Based on preliminary data from the Bioresource, we anticipate recruiting approximately 2500 cases of respiratory tract infection, 2000 cases of urinary tract infection syndromes and 1000 cases with bacteraemia.

The Bioresource has been established to address a range of research questions from discovery science through the clinical trials and consequently we have not stipulated a single outcome measure. We will first summarise the clinical and epidemiological characteristics of the cohort, specifying the primary outcome as the proportion of individuals in whom a microbial diagnosis is achieved, stratified by clinical infection syndrome (respiratory, urinary tract, skin and soft tissue, systemic with no foci of infection). Secondary outcomes will include: estimating the sensitivity, specificity, positive and negative predictive values of a syndromic diagnosis of infection in the ED compared to diagnostic coding at discharge from hospital; identification of clinical and epidemiological factors associated with adverse outcomes including length of stay, admission to intensive care, death; and predictors of bacteraemia. Future studies based on data and/or samples from this cohort will be required to submit a full proposal and analysis plan before being granted access to data and/or samples.

### Patient and public involvement

The BioAID protocol was developed with advice from the UCL Partnership Public Engagement Patient Panel. This group provided particular input on the issue of deferred consent and have continued to be involved as members of the BioAID Advisory Board.

### BioAID governance

BioAID is overseen by an executive committee comprising the PI’s from each participating site, and meets quarterly to review progress and recruitment and to process applications for access to data and samples.

### Ethical approvals

A specific aim of BioAID is to obtain RNA samples before treatment commences because transcriptional profiles can be modified by antimicrobial or other treatments and there is increasing interest in host responses as part of diagnosis in infection ^14– 16^. Given the clinical necessity to initiate treatment for patients urgently (typically within 1–2 hours), and the fact that patients may have impaired consciousness or be distressed, it is rarely possible to obtain genuine informed consent from patients before collecting blood for RNA and, should it be possible, there would be significant biases in the patients recruited. Ethical approval and section 251 exemption have therefore been obtained for BioAID researchers to seek deferred consent from patients from whom a RNA specimen has been collected (or from their relatives/nominated consultee) within 72 hours of blood sample collection (REC ref: 14/SC/0008).

### Governance, data protection and data management

The BioAID dataset is pseudo-anonymised. Participants are allocated a unique identification number (UIN) and at each participating site a separate electronic and hard copy file is maintained linking the UIN with the patient’s hospital number, other identifiers and contact details. The local Principal Investigator has access to the linkage codes. The live BioAID database is held within the NHS firewall.

### Data management and access

The BioAID database will be curated by the research team. Histograms will be plotted to investigate the distribution of continuous variables and rules will be applied to identify likely outliers based on laboratory reference ranges and errors in dates and age. Samples collected through the Bioresource and associated meta-data will be made available to researchers worldwide. To qualify for access, an application form including a research protocol should be submitted to the study coordinator for consideration by the BioAID Executive Committee ( m.noursadeghi@ucl.ac.uk). Interested researchers are expected to cover the processing costs of sample aliquots from the Bioresource. Access to samples will be subject to a material transfer agreement. All studies using BioAID data and samples will be required to submit annual reports to the Executive Committee and a copy of all the derived data must be deposited within the BioAID database. Publications arising from use of the Bioresource are expected to acknowledge the support of the NIHR Biomedical Research Centres and to recognise BioAID investigators.

## Dissemination of findings

Anonymised data will be made available at the time of peer-reviewed publications, or by 12 months after completion of the project. Raw sequencing, genotyping data and linked metadata will be made available through quality controlled public repositories to maximise their use by the scientific community. Specifically, European Bioinformatics Institute Array Express repository, for genome-wide transcriptomic data, and the European Bioinformatics Institute Genome-Phenome archive for genotypic and phenotypic data. Processed and analysed data sets will also be made available through supplementary on-line content associated with peer-reviewed scientific publications. All new computational analysis software that we develop in the course of this project will made publicly available on the Bioconductor platform. Research findings will be communicated to the scientific community via open access peer reviewed publications and presentation at conferences. BioAID investigators will work with the UCL-Partnership Public Engagement Patient Panel to disseminate research findings to patients and the public.

## Study status

Ethical approval was granted for BioAID in February 2014 and recruitment began shortly afterwards. To date, > 4000 participants have been recruited across two NHS Trusts; a third site will join in 2018.

## Conclusions

The purpose of BioAID is to support large number of collaborative projects and associated research publications. To date, BioAID has been used primarily for the development and validation of transcriptomic gene signatures for bacterial infection ^7^, but the sample collection also provides unprecedented opportunities to evaluate proteomic and metabolomic biomarkers. In the future, there is scope to use BioAID as a recruiting framework for inpatient clinical trials or as a means of identifying candidates for studies investigating host susceptibility to infection or host-pathogen interactions. As the number of sites participating in BioAID increases it is anticipated that there will be a range of applications to use this dataset.

## Data availability

No data are associated with this article.

## Supplementary material

Supplementary File 1 - Case reporting form.
