# Supplementary material for: Cohort study protocol: Bioresource in Adult Infectious Diseases (BioAID)
Source: Wellcome Open Res. 2018 Aug 8;3:97. [Version 1] doi: 10.12688/wellcomeopenres.14690.1 (PMC6171553; doi:10.12688/wellcomeopenres.14690.1)
Supplement: Supplementary file 1 [file wellcomeopenres-3-15996-s0000.tgz › 44c8e0e8-bf10-4273-9d69-1f1412e12409.pdf]

BioAID CRF

**Hospital No.**		**Study UIN**	
**BioRes (Y/N)**		**BioRes Sticker**	

Demographics

**DOB**		**Ethnicity (Main)**	
**Gender**		**Ethnicity (Sub)**	

On Admission

**Presentation Date**		**Consent Date**	
**Duration of Illness**		**Preadmission Antibiotics**	
**Readmission <1 Week**	*Y* *N*		

**RR**		**O_2 Sats**	*AIR* *O_2*	**%**	**HR**		**BP**	**/**	**Temp**		**A V P U**

Differential Diagnosis

**1st System**		**1st Syndrome**	
**2nd System**		**2nd Syndrome**	
**Antibiotics on Admission**		**Route**	
		**Route**	
		**Route**	

Samples Collected

	**BioAID**				**BioRes**			**BioAID**	
**Timepoint**	**T1 RNA**	**T1 Serum**	**T2 RNA**	**T2 DNA** (If no BioRes)	**CAT**	**4ml EDTA**	**10ml EDTA**	**TM**	**Isolate**
**Date Collected**									

Outcome

**Discharge Date**			
**Discharge System**		**Discharge Syndrome**	
**Adverse Outcome**		**Outcome (Other)**	

Risk Factors

- ○ Alcohol misuse
- ○ Autoimmune Rheumatological Disease
- ○ Auto-inflammatory Disease
- ○ Bone Marrow Transplantation
- ○ Chronic Heart Disease
- ○ Chronic Kidney Disease
- ○ Chronic Liver Disease
- ○ Chronic Lung Disease
- ○ Chronic Neurological Disability
- ○ Developing World Travel <3 months
- ○ Diabetes
- ○ Genetic blood disorder
- ○ Haematological Malignancy
- ○ HIV
- ○ Hospital Admission <1 year
- ○ Immunosuppression (other)
- ○ Immunosuppression with Corticosteroids
- ○ Indwelling Intravascular Catheter
- ○ Indwelling Urinary Catheter
- ○ Infectious Diseases Contact
- ○ Inflammatory Bowel Disease
- ○ Intravascular Prosthesis or Stent
- ○ IVDU
- ○ Medical device implant
- ○ Occupational Animal Exposure
- ○ Occupational Water Exposure
- ○ Orthopaedic Prosthesis
- ○ Pregnancy
- ○ Previous Resistant Bacterial Isolate
- ○ Prosthetic Heart Valves
- ○ Solid Organ Malignancy
- ○ Solid Organ Transplantation
- ○ Surgery <3 months
- ○ Transplantation
- ○ Other: ……………………………………………………………
  ………………………………………………………………………
  ………………………………………………………………………
- ○ NA

Name: ………………………………………………… Signature ………………………………………… Date: ……… /…….. ./…………
Section(s) Completed:

Name: ………………………………………………… Signature ………………………………………… Date: ……… /…….. ./…………
Section(s) Completed:

Name: ………………………………………………… Signature ………………………………………… Date: ……… /…….. ./…………
Section(s) Completed:
