# Supplementary material for: Cohort study protocol: Bioresource in Adult Infectious Diseases (BioAID)
Source: Wellcome Open Res. 2018 Aug 8;3:97. [Version 1] doi: 10.12688/wellcomeopenres.14690.1 (PMC6171553; doi:10.12688/wellcomeopenres.14690.1)
Supplement: Peer review history

# Peer review: Referee response for version 1

**DOI:** 10.21956/wellcomeopenres.15996.r33835

**Published:** 2018-10-01

**Authors:** Thomas Evans

**Affiliations:** 1 Institute of Infection, Immunity and Inflammation, University of Glasgow, Glasgow, UK

This article describes a cohort study protocol to establish a bioresource in adult infectious disease. The study aims to obtain deferred consent for collection of clinical samples and phenotypic data on patients admitted with suspected infection. The aim is to provide a bioresource of high-quality phenotypic data that can be linked to the patients’ samples. This would then be available for the research community to access, allowing investigations  into novel biomarkers and diagnosis for infections, as well as defining risk factors, pathogenic mechanisms and detailed epidemiology of a variety of infection syndromes.

 The authors set out in detail the aims, rationale and methods that are in place for this ongoing study. The outlined protocol is well thought out and sufficient to meet the aims of the study. Ethical permissions are in place. The intention is to recruit any patient with a suspected infection – this broad approach will ensure a range of infection syndromes are studied, although large sample sizes will be required to ensure adequate power for individual syndromes such as, say, urinary infection. One major criticism is the lack of a control group. Inflammatory responses to infection will overlap with similar responses in a variety of other conditions. However, I think the idea is to use the data collected as a ‘discovery’ resource and that future work based on data from this study would then go on to test the validity of, say, a novel biomarker in a specific prospective trial. It would be helpful if the authors could expand on considerations of a control group and what future work might thus be required for validation of results.

I have read this submission. I believe that I have an appropriate level of expertise to confirm that it is of an acceptable scientific standard.

# Peer review: Referee response for version 1

**DOI:** 10.21956/wellcomeopenres.15996.r33660

**Published:** 2018-09-20

**Authors:** Emma Thomson

**Affiliations:** 1 MRC-University of Glasgow Centre for Virus Research, 464 Bearsden Road, Glasgow, G61 8QQ, UK

The BioAID resource provides a much needed biocollection of well-curated prospectively collected biological samples from patients presenting to one of several large teaching hospitals with a suspected infection syndrome. Importantly, samples will be obtained at presentation alongside blood cultures often prior to the administration of antimicrobial therapy.

 It aims to provide researchers with access to large numbers of samples providing a route to meaningful studies of biomarker identification, host genetics and pathogen identification. The scale of the resource will mean that genetic associations with outcome will be sufficiently powered for meaningful interpretation.

 Judging by the listed authors, the design and the stated broad aims, the current focus appears to be to address bacterial infection, initially aiming to identify 2500 cases of respiratory infection, 2000 cases of UTI and 1000 cases of bacteraemia. However, the potential for identification of other pathogens, for example viral pathogens is also present.

**Minor suggestions**

1. Unfortunately, the case reporting form (Supplementary Figure 1) is not available for review (weblink error) but is likely to provide essential follow-up information on patient demographics and symptoms as well as outcome. I assume it will also include a travel history.
2. While it is clear that “microbial isolates and sera obtained from the patient during admission” will be retrieved, there might also be an opportunity to gather other relevant samples e.g. CSF or urine which could be used for pathogen discovery at a later date. Sputum and respiratory samples from the 2500 cases of respiratory infection would allow a search for viral as well as bacterial pathogens and identification of co-infections.
3. The ethics for the collection of samples is carefully considered and appropriate with prospective collection and retrospective approval (in the case of death by the next-of-kin). I wonder, however, if the possibility of returning results to the relevant patient has been discussed. I am referring, in particular, to HIV (and other chronic viral infections such as HCV and HTLV) which are likely to be identified if RNA sequencing is planned. There may be a role for returning such a result to the patient following clinically validated testing.

**Summary**

 In summary, this is an exciting, large-scale, unique and much needed bioresource with the potential to unite advances in metabolomics and genomics with clinical pathogen diagnosis, host susceptibility and identification of predictive biomarkers of disease. It is highly likely to provide a platform for the development of multiple meaningful studies and will facilitate the introduction of technological advances into the health service to directly improve patient care.

I have read this submission. I believe that I have an appropriate level of expertise to confirm that it is of an acceptable scientific standard.
